# A Rare Presentation of Adult Intussusception

**DOI:** 10.7759/cureus.91419

**Published:** 2025-09-01

**Authors:** Talha Wahid, Monica Paneru, Andrea Escalante, Kira B Fenton

**Affiliations:** 1 Dr. Kiran C. Patel College of Osteopathic Medicine, Nova Southeastern University, Fort Lauderdale, USA; 2 Internal Medicine, Broward Health Medical Center, Fort Lauderdale, USA

**Keywords:** adult intussusception etiology, ct target sign in intussusception, enteroenteric vs. colonic intussusception, intussusception, intussusception in adults, short-segment intussusception management, small bowel intussusception, spontaneous resolution of intussusception

## Abstract

Intussusception occurs when one segment of the intestine telescopes into another, potentially causing bowel obstruction and impaired blood flow. While more commonly observed in children, adults may also be affected. In most adult cases, an underlying cause such as a neoplasm, adhesion, or inflammatory lesion is identified. However, rare cases present without a clear precipitating factor. We report a case of a 40-year-old male with a history of constipation and internal hemorrhoids who presented with diffuse abdominal pain and multiple episodes of non-bloody, non-bilious vomiting. He denied recent illness, travel, or family history of gastrointestinal disease. Physical examination revealed diffuse tenderness without peritoneal signs, and laboratory results were unremarkable. A non-contrast CT scan of the abdomen and pelvis revealed an enteroenteric intussusception without evidence of bowel obstruction, mass, or ischemia. Given the patient's stable clinical status and lack of high-risk features, conservative management was initiated. This included intravenous fluid hydration, bowel rest (NPO), antiemetic therapy, and close monitoring with serial abdominal examinations. A small bowel follow-through with oral contrast later confirmed spontaneous reduction of the intussusception. The patient’s symptoms resolved, and he was gradually reintroduced to oral intake. He remained stable throughout hospitalization and was discharged in good condition with outpatient follow-up arranged. This case demonstrates that conservative treatment may be appropriate for adult intussusception in select cases, particularly when there are no signs of obstruction, ischemia, or a clear lead point. Timely imaging and close clinical assessment are critical to guide management and avoid unnecessary surgical intervention.

## Introduction

Intussusception occurs when a proximal segment of the intestine invaginates or 'telescopes' into a distal segment, causing obstruction and compromised blood flow. It primarily affects children between six months and five years old; however, intussusception can occur in 1% to 5% of adults [1]. It is typically associated with an identifiable cause in 90% of cases, such as a neoplasm, adhesion, or inflammatory lesion [2]. Here, we report a case of intussusception in an adult with no obvious underlying pathology or medical history.

## Case presentation

**Figure 1:**
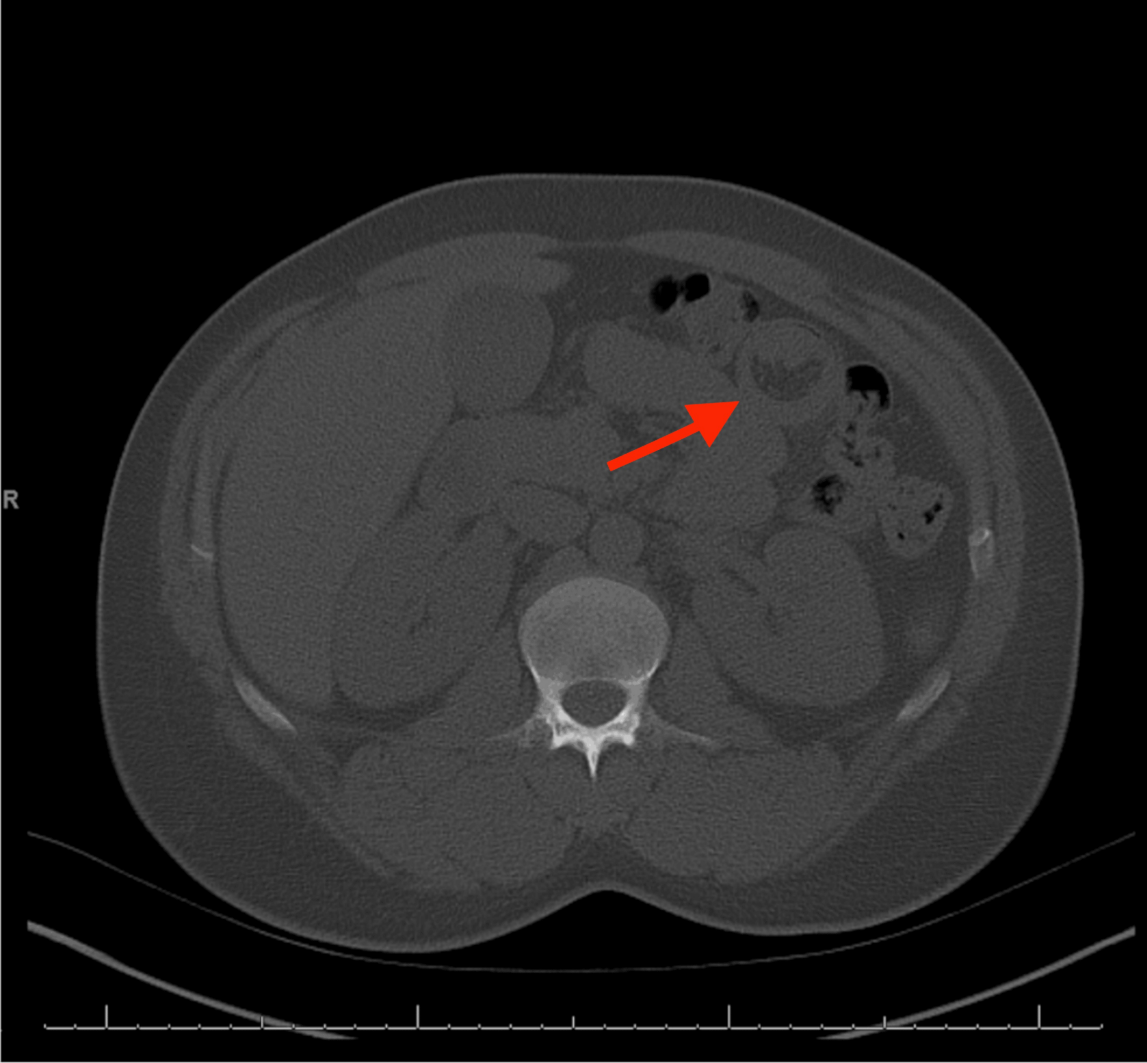
Axial CT scan of the abdomen without contrast demonstrating a 'target' appearance consistent with enteroenteric intussusception (red arrow) No evidence of obstruction, mass lesion, or ischemic changes was observed.

A 40-year-old male with a history of constipation and internal hemorrhoids presented to the emergency department with a two-day history of diffuse, non-radiating abdominal pain that was 8/10 in severity. His symptoms included 14 episodes of clear, non-bloody, non-bilious vomiting. He reported regular bowel movements without melena or hematochezia and denied fever, chills, diarrhea, chest pain, or shortness of breath. He reported no recent sick contacts, travels, or hospitalizations. He denied any previous colonoscopies or endoscopies. His social history was significant for smoking one cigarette and marijuana daily, consuming four shots of alcohol weekly, and following a fatty diet. He denied any significant family history of gastrointestinal cancer or inflammatory conditions. On initial physical evaluation, he had diffuse abdominal tenderness without peritonitis or palpable lumps. The patient’s stature was also within normal limits, and there were no mucocutaneous pigmentations (such as peri-oral or lip lesions). Laboratory results were unremarkable, and non-contrast CT scans of the abdomen and pelvis revealed an enteroenteric intussusception. No masses or signs of bowel ischemia were noted (Figure 1).

**Figure 2:**
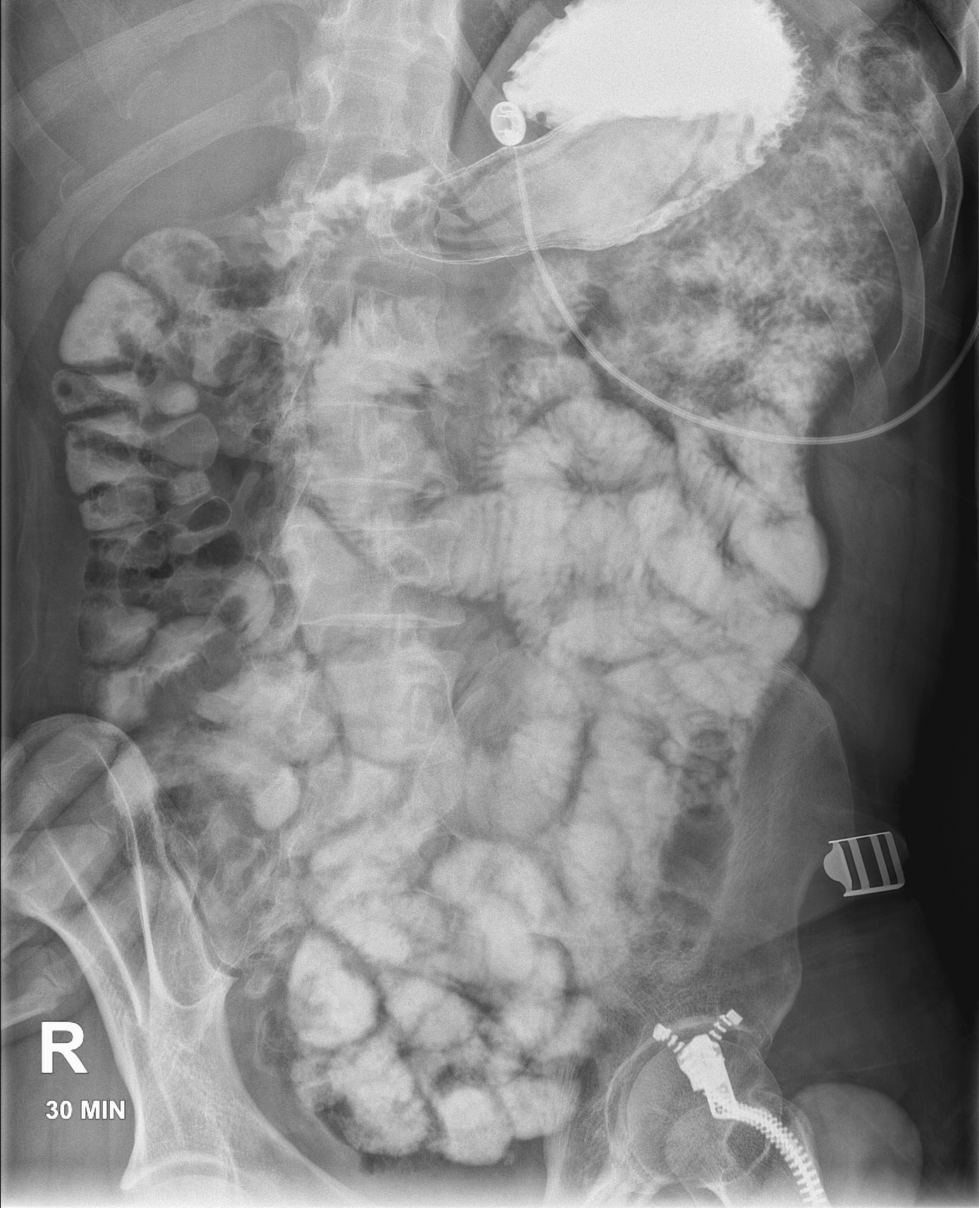
Small bowel series demonstrating that the intussusception has cleared

Given the patient's clinical stability and the absence of bowel obstruction or ischemia, conservative management was pursued. This included intravenous fluid hydration, bowel rest (nil per os (NPO) or nothing by mouth), antiemetic therapy with Zofran, and close monitoring with serial abdominal examinations. A small bowel series with oral contrast revealed spontaneous reduction of the intussusception (Figure 2). The patient tolerated the diet well and was instructed to follow up as an outpatient with a gastroenterologist for further evaluation. 

## Discussion

Adult intussusception is a rare entity, accounting for only 1% to 5% of all cases of intussusception and approximately 0.003% to 0.02% of all hospital admissions [1,3]. In children, intussusception is most commonly ileocolic, with acute colicky abdominal pain, currant-jelly stools, and a palpable mass. In adults, enteroenteric or colonic sites predominate, with more nonspecific, subacute symptoms such as abdominal pain, nausea, and vomiting. This difference explains the diagnostic challenge in adults [4]. And unlike pediatric cases, which are mostly idiopathic, adult intussusception is often secondary to an underlying pathology in 70% to 90% of cases [2,5]. Common causes include malignancies (both benign and malignant tumors), postoperative adhesions, inflammatory bowel disease, Meckel’s diverticulum, and post-surgical changes [4,5]. In the absence of an identifiable lead point, transient and self-resolving cases have been reported, usually in association with dietary habits, intestinal dysmotility, or idiopathic etiologies [3,4]. Therefore, since the patient had no prior abdominal surgery, no family history of GI malignancy, and no exam or imaging evidence of mass or inflammation, this case can be classified as the rarer idiopathic form.

Clinical presentation and diagnosis

As explained, the clinical presentation of intussusception in adults is often nonspecific, making diagnosis challenging. Chronic or subacute presentations can mimic other conditions such as partial bowel obstruction, irritable bowel syndrome, or malignancy [5]. Therefore, non-contrast CT is the gold standard for diagnosis, with an accuracy rate exceeding 90% [5]. The classic 'target' or 'sausage-shaped' mass appearance on CT imaging is highly suggestive of intussusception [5]. An MRI may also be useful, particularly in cases requiring further evaluation of soft tissue involvement. Additionally, ultrasound may be utilized, though it is more commonly used in pediatric cases [5].

Management considerations

Management of adult intussusception depends on the underlying cause, severity, and location [4,6]. In cases involving a lead point, surgical resection is typically required due to the high risk of malignancy, especially in colonic intussusception, where malignancy is identified in up to 65% of cases [2,3]. However, in enteroenteric intussusception without evidence of a lead point, conservative management may be an option, as demonstrated in this case [5].

Short-segment intussusceptions (<3.5-3.8 cm) are more likely to resolve spontaneously and may be managed with observation, serial imaging, and supportive care [3]. Bowel rest, intravenous fluids, and nasogastric decompression may be beneficial in symptomatic cases [6]. In contrast, longer-segment intussusceptions, particularly those causing bowel obstruction or ischemia, often require surgical intervention [4,7]. Contrast enemas, such as barium or gastrografin, have shown utility in both diagnosis and reduction in some cases [5]. Although conservative management is an option in selected stable patients with transient, short-segment intussusception and no lead point. Surgical management remains standard when pathology is suspected. This case illustrates a scenario where conservative management was safe and successful.

## Conclusions

The prognosis of adult intussusception largely depends on the underlying cause. In cases without an identifiable lead point, outcomes are generally favorable with conservative management. However, recurrence remains a possibility, necessitating close follow-up and further investigation, particularly with endoscopy, to rule out underlying pathology. Colonoscopy or small bowel enteroscopy may be warranted in cases of recurrent symptoms or persistent suspicion of malignancy.

This case highlights the importance of recognizing adult intussusception as part of the differential diagnosis for abdominal pain and vomiting. The spontaneous resolution observed in this patient underscores the role of conservative management in select cases. Further research is needed to better characterize risk factors for idiopathic intussusception in adults and to optimize management strategies for these patients.
